# Heterogeneity and optimal study design between cell lines in induced pluripotent stem cell-based cardiac disease modeling

**DOI:** 10.1093/stcltm/szag001

**Published:** 2026-01-28

**Authors:** Renee G C Maas, Chris Denning, Joost P G Sluijter

**Affiliations:** Utrecht Regenerative Medicine Center, Circulatory Health Research Center, University Utrecht, Utrecht, 3584 CS, The Netherlands; Experimental Cardiology Laboratory, Department of Cardiology, Division Heart and Lung, University Medical Center Utrecht, Utrecht, 3584 CX, The Netherlands; Department of Stem Cell Biology, Biodiscovery Institute, University of Nottingham, Nottingham, NG7 2RD, United Kingdom; Utrecht Regenerative Medicine Center, Circulatory Health Research Center, University Utrecht, Utrecht, 3584 CS, The Netherlands; Experimental Cardiology Laboratory, Department of Cardiology, Division Heart and Lung, University Medical Center Utrecht, Utrecht, 3584 CX, The Netherlands

**Keywords:** hiPSC, cardiomyocytes, heterogeneity, study design, cardiac *in vitro* model

## Abstract

Human-induced pluripotent stem cell (hiPSC) technologies have provided access to *in vitro* models of inaccessible human cardiomyocytes (CMs), providing new insights into human disease mechanisms, therapy strategies, and cardiac toxicology. However, the robustness of reproducible outcomes and integration of data among research groups are hampered due to the variation between cell lines, clones, and batches-to-batch differences. These variable outcomes in hiPSC models are caused by differences in human donors, genetic stability, and experimental variability, which affect morphology, cellular heterogeneity, transcript and protein abundance, and differentiation potency. This review summarizes the usage of hiPSC-CMs obtained from multiple lines and evaluates the corresponding experimental variation between studies to perform in-depth *in vitro* power calculations. Our meta-analyses show that although 4 or more hiPSC lines are used in 21 published case-control studies, these reports still contain high heterogeneity between functional parameters. In specific CM readouts, the SD is >40%, meaning that the variation between different cell lines is larger than the effect of the studied mutation, drug response, or toxicity. Results indicate a need for careful selection of hiPSC lines, controls, and readout stability and these insights will further guide the power of hiPSC lines in biomedical applications.

## Graphical abstract

**Graphical Abstract:**
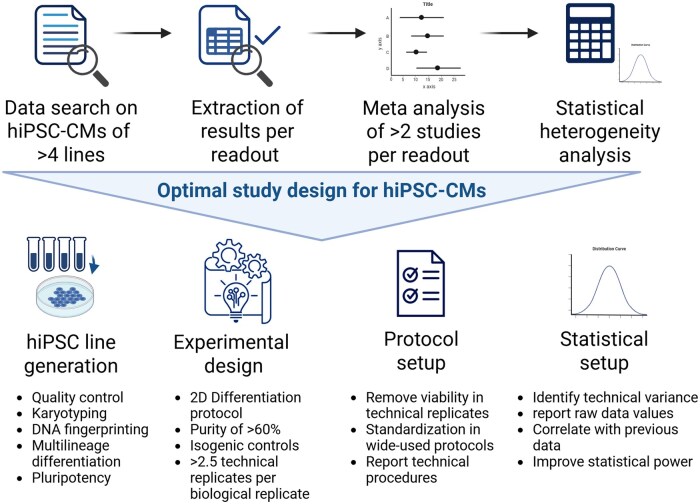

Significance statement Biological heterogeneity is an inherent feature of human physiology and represents both a challenge and an opportunity in cardiovascular research. However, many human-induced pluripotent stem cell (hiPSC)–based cardiovascular studies to date rely on limited cohort sizes, potentially underestimating the extent and impact of biological variability. By reviewing 21 studies that assessed cardiac phenotypes across more than 4 hiPSC lines, this work highlights substantial baseline heterogeneity across electrophysiological, contractile, morphological, and metabolic readouts, even among commercially available cardiomyocyte lines generated using standardized protocols. Notably, variability in action potential duration, contractile force, and cellular metabolism often exceeds physiological ranges observed in the human heart. These findings underscore the critical importance of incorporating adequately powered, multi-line study designs and rigorous quality control strategies in hiPSC-based cardiovascular research. Understanding and addressing the sources of this heterogeneity are essential for improving reproducibility, enhancing disease modeling accuracy, and enabling reliable translational research.

## Introduction

Advancement of human-induced pluripotent stem cell (hiPSC) technologies enables almost limitless numbers of hiPSC-derived cardiomyocytes (hiPSC-CMs) to be produced, including from patients suffering from genetic conditions such as arrhythmogenic cardiomyopathy, dilated cardiomyopathy (DCM), ­hypertrophic cardiomyopathy (HCM), non-compaction cardiomyopathy, and lysosomal storage disorders.^1-3^ The hiPSC technology could not only serve as a proxy for clinical trials but also have no limitation in its use for the total number of different “people” that could be used for, for example, drug screenings. The massive expansion and biobanking^4^ and the generation of 3D microtissues in a 96-well and 384-well format offer great potential for high-throughput (HT) disease modeling and subsequent screening of therapeutic interventions. The efficient development of hiPSC-CM models from any cardiomyopathy and any patient can, now more than ever, be used as “clinical trials in a dish” (CTiD).^5^ The development of a newly discovered drug to clinical implementation can take more than 10 years, with costs of approximately 2 billion USD for the whole process. This disappointing reality of promising preclinical findings failing in 89% of the studies from animal models into effective therapies raised serious concerns within the scientific community.^6^ The recent FDA Modernization Act 2.0 has opted for alternative methods such as hiPSC to bolster the preclinical data pipeline, aiming to reduce the dependence on animal models.^7^ The combination of bioengineered 3D techniques with hiPSC technology is currently strongly suggested to improve our preclinical-to-clinical trial pipeline for new therapeutics.

In statistical sample size estimation studies, the probability of an event can be calculated, which is the probability that the outcome of the experiment is contained in the event.^8^ Here, a sample size of 4 different cell lines demonstrates the drug toxicity/sensitivity probability of the observations in 34%,^9^ whereas 22 individual lines, roughly the number of individuals in the phase I clinical trial, would achieve a 90% probability of predicting events that occur in 10% of the population. With 250 lines, the assay could predict events in 1% of the population with a 90% probability.^10^ The relative costs of these hiPSC-derived microtissues are very low, resulting in the cost for a screening of 22 lines with 5 drug concentrations and 5 replicates per condition to be under 200 euros (±0.22 per spheroid).^11^ This enables big pharma to reduce the costs of drug development by performing drug screenings that have the intrinsic potential for a higher likelihood to remove unsafe and ineffective new drugs from their pipelines while continuing the development of drugs that are safe for human administration. These phase II–like CTiDs could follow the same principles as a clinical trial, with similar donor cohort design, scale, and confidence in the prediction.

However, few studies have thus far used 4 or more individual hiPSC-CMs for the prediction of disease modeling, drug response, or cardiac toxicity. Since many lines use a maximum of 3 lines, the effect size of 4 or more hiPSC lines has not been studied often in cardiovascular research. In this systematic review with subsequent meta-analysis, we summarize study outcomes and the corresponding variability of CMs derived from 4 or more hiPSC lines for optimal study design and to generate statistical power.

## Research

We compared commonly used study designs for hiPSC-based disease modeling, drug response, or cardiac toxicity in terms of applicability for different research questions, statistical analysis, and attainable power. First, the assessment and outcome per study type were summarized in Table 1. We also listed the somatic cell source, differentiation protocol, and coating strategy used in the studies (Table S1). The somatic source varied much, still mainly fibroblasts or peripheral blood mononuclear cells (PBMCs) were used for the hiPSC reprogramming. Next, 7 studies used embryonic body (EB) formation, 10 studies a form of monolayer (2D) differentiation, and in 5 studies, the hiPSC-CMs were commercially produced without specified protocols. Interestingly, 2 studies used multiple differentiation protocols in their study to produce hiPSC-CMs. The majority of listed studies (12) used Matrigel for the coating and culturing of hiPSC-CMs, whereas gelatin (6) and fibronectin (2) were also used.

**Table 1.** Overview of all studies using 4 or more hiPSC lines for disease modeling, drug response, and cardiac toxicity *in vitro*.

Study type	hiPSC lines (number)	CM age (days)	Analysis	Assessment	Outcome	Ref.
**Disease modeling**	RYR2 (6), TPM1 (2), MYBPC3 (2), KCNQ1 (2), HERG (4), LMNA (2), healthy subjects (2)	∼30-50	Calcium transients by machine learning	Analysis of 12 variables in calcium peaks for the separation of disease vs control	Efficient classification accuracy of 87% between the disease group and controls.	[12]
**Disease modeling**	MYBPC3 (4), healthy subject (1)	13	Morphology, gene/protein expression	Splicing alteration analysis	Study intronic variation in splicing abnormalities of MYBP3 variants.	[13]
**Disease modeling**	DMD (4) healthy subjects (2)	∼20	LC–MS, patch clamp, EM, confocal microscopy	Study if DMD hiPSC-CM are associated with metabolic deficits	DMD hiPSC-CMs recapitulated to some extent the disease phenotypes morphologically and functionally, and metoprolol improved myofilament organization	[14]
**Disease modeling**	TNNT2 (4), probands (3)	20-50	Sarcomere distribution, MEA, patch clamp, calcium, contractility analysis	Analyze functional properties, describe the potential underlying etiology, and test metoprolol	Impairment in myofilament regulation, Ca2+ handling, and force production of individual CMs, explain DCM clinical phenotype	[15]
**Disease modeling**	MYH7 (5), probands (5)	20-60	Optical Ca2+ imaging, MEA, patch clamp, confocal microscopy, single-cell qPCR	Elucidate mechanisms underlying HCM and test pharmacological restoration	MYH7 hiPSC-CMs recapitulate HCM phenotype *in vitro*. The pharmacological screening of 13 drugs, revealed that drugs blocking Ca2+ and Na2+ proved to be most efficient to restore beating frequency. Verapamil prevented hypertrophy in the MYH7 hiPSC-CMs	[16]
**Disease modeling**	Healthy subjects (6)	30	Overall distribution of action potential	Variability of arrhythmias per cell line, per differentiation protocol and batch	Even the same cell line and differentiation protocol reveals variability, indicating the importance of modeling arrhythmias in hiPSC-CMs.	[17]
**Disease modeling**	LMNA variants (7), healthy subjects (3)	∼40	Morphology, calcium transients, gene expression	Variability in specific LMNA mutation sites and types.	Mechanistic and phenotypic heterogeneity in LMNA expression, kinase pathway activation, sarcomere disarray, arrhythmias, and ion channel gene expression.	[18]
**Disease modeling**	Healthy subject (1), iTTN (4)	∼40	Contractility, morphology, calcium transients	Variability in TTN isoform mutation sites and types.	Mechanistic and phenotypic heterogeneity in sarcomere formation, contraction, and calcium handling.	[19]
**Disease modeling**	MYBPC3 (1), MYH7 (4), DMD(3), TNNT2(3), TTN(2), healthy subjects (5)	30	Telomere shortening	Study telomere shortening as a hallmark of genetic HCM and DCM.	HCM and DCM CMs exhibit significant telomere shortening relative to healthy controls.	[20]
**Drug response**	Healthy subjects (10)	∼50 (EHTs)	Contractility, AP, morphology, gene expression	Screening of 7 inotropic indicator compounds.	Baseline phenotypes of healthy control cell lines differ considerably, drug responses were qualitatively similar.	[21]
**Drug response**	RyR2 (6)	∼30-50	Calcium transients by machine learning	Antiarrhythmic effect of dantrolene after adrenaline administration	Classification accuracy of 65.6% and sensitivities (true positive rates) of 79.7% for responders	[22]
**Drug response**	Healthy subjects (6)	∼25	Qualitative differences in action potentials	Eight different classes of pharmacological reagents	hiPSC-CMs from female donors were more sensitive to dofetilide/cisapride. Male donors were less sensitive to the 2 typical hERGs. One line did not show a chronotropic effect to Isoproterenol. Two lines reacted to tetrodotoxin at very low concentrations and were more sensitive to external stimulation.	[23]
**Drug response**	Healthy subjects (5)	∼25	Calcium transients, gene expression, patch clamp	Screening of 10 proarrhythmic compounds. Bayesian statistics analysis was used.	All cell lines exhibited expected CTD elongation/shortening. Various responsiveness in 2 lines; no response to Quinidine but highly to moxifloxacin.	[24]
**Cardio-toxicity**	Healthy subjects (6)	27-29	Viability, calcium transients, gene expression	Screening of 4 TOP2 inhibitors and Trastuzumab.	TOP2i induces cell death and affects calcium handling and gene expression levels. Trastuzumab does not.	[25]
**Cardio-toxicity**	Healthy subjects (10)	∼40	Morphology, MEA array (FPD), RNA microarray	Risk of moxifloxacin-induced long QT in patients vs. hiPSC-CMs	Significant correlation of FDP response to the inter-individual differences observed in vivo	[26]
**Cardio-toxicity**	Healthy subjects (14)	∼50	MEA array (FPD), patch-clamp analysis, RNAseq	Risk prediction in hiPSC-CMs from patients with low (7) or high (7) sensitivities to Sotalol-induced long QT	Strong correlation of FDP response to the inter-individual differences observed in vivo	[27]
**Cardio-toxicity**	Healthy subjects (16)	∼30	APD recordings (CellOPTIQ)	Risk of dofetilide or moxifloxacin-induced long QT in patients vs. hiPSC-CMs.	No significant correlation between the in vitro hiPSC-CM and the clinical response of the same subject	[28]
**Cardio-toxicity**	Healthy subjects (11)	∼40	Calcium, contractility, morphology, phosphorylation arrays	Cardiac safety index screen of 21 chemotherapeutic kinase inhibitors	Good correlation between in vitro cardiotoxicity and the clinical incidence of cardiotoxicity	[29]
**Cardio-toxicity**	Healthy subjects (27)	∼40	Ca2+ flux assay, cytotoxicity, TempO-Seq, mitochondrial high-content cell imaging	Feasibility of hiPSC-CMs as population- based in vitro model for inter-individual variability	hiPSC-CMs can be used to characterize inter-individual responses in untreated and chemical-treated hiPSC-CMs	[30]
**Cardio-toxicity**	Homozygous HLA donors (13)	48	Viability screening, MEA array, TUNEL	Screening of 2375 clinically approved compounds for cardiotoxicity	hiPSC-CM screening confirms known cardiotoxic compounds and identifies several unknown cardiotoxic compounds.	[31]
**Cardio-toxicity**	Covid (13)	n/r	Immunofluorescence	Cardiotoxicity of the current and prospective COVID-19 treatment options.	Cardiotoxicity was observed in remdesivir and arbidol-treated cells in most population-representative lines.	[32]

Abbreviations: APD, action potential duration; CTD, calcium time duration; DCM, dilated cardiomyopathy; DMD, Duchenne muscular dystrophy; EHTs, engineered heart tissues; EM, electron microscopy; FPD, field potential durations; HCM, hypertrophic cardiomyopathy; hERGs, human ether-à-gogo–related gene; HLA, human leukocyte antigens; KCNQ1, potassium voltage–gated channel subfamily Q member 1; LC–MS, liquid chromatography–mass spectrometry; LMNA, lamin A/C; MEA, multielectrode array; MYBPC3, myosin-binding protein C; MYH7, myosin heavy chain 7; n/r, not reported; RYR2, Ryanodine Receptor 2; TNNT2, cardiac troponin T; TOP2 inhibitors, topisomerase 2 inhibitor; TPM1, tropomyosin 1; TTN, titin.

**Figure 1.**
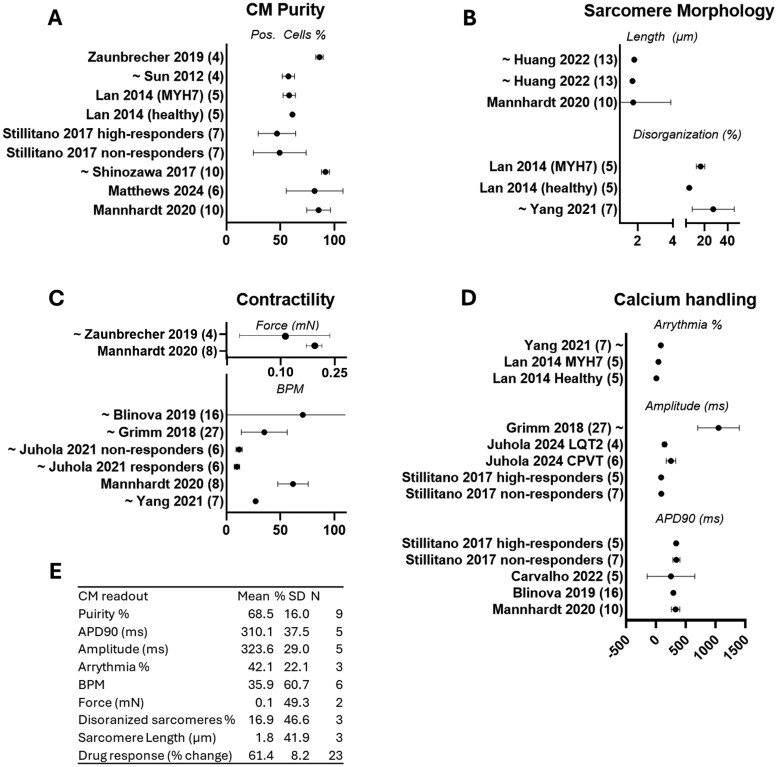
Cardiac readouts and technical variation per study. (A) Meta analysis of CM purity % in 9 studies. (B) Meta analysis of sarcomere morphology consisting of sarcomere length (µM) in 3 studies and % of sarcomere disorganization in 3 studies. (C) Meta analysis of contractility consisting of force (mN) and 6 beats per minute (BPM) studies. (D) Meta analysis of calcium handling, consisting of arrhythmia %, 3 studies, amplitude (ms) 5 studies, and action potential duration 90 (APD, ms) in 5 studies. Numbers between brackets indicate hiPSC lines used per study. (E) Summarizing table with the mean values, percentage of variation (%SD), and *N* = number of hiPSC lines per readout.

**Figure 2.**
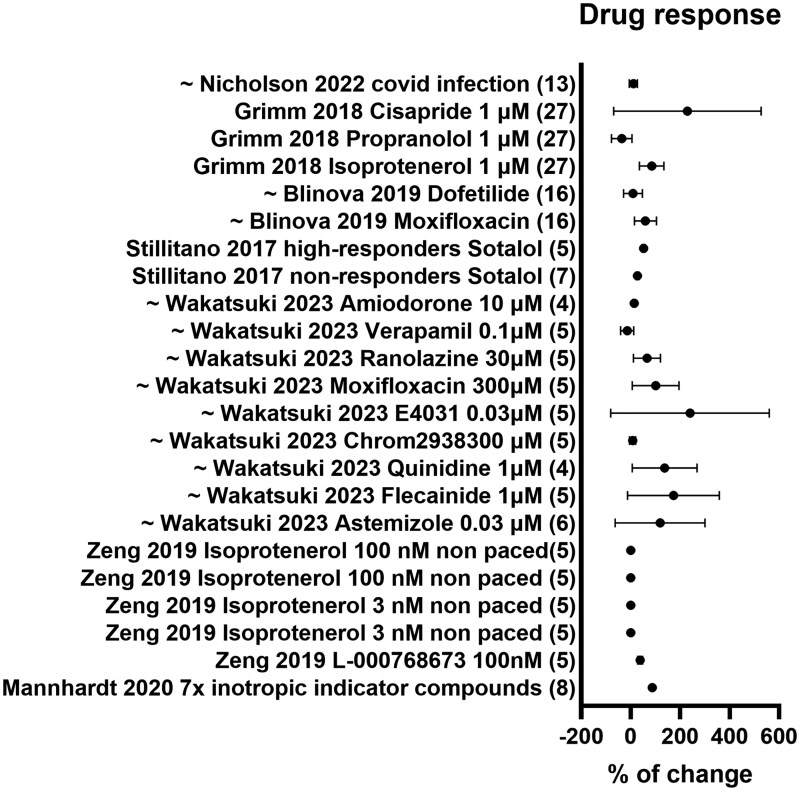
Drug response in hiPSC-CM studies. Meta analysis of the drug response effect in 24 conditions, with the percentage of change toward baseline used as functional parameter. Numbers between brackets indicate hiPSC lines used per study.

**Figure 3.**
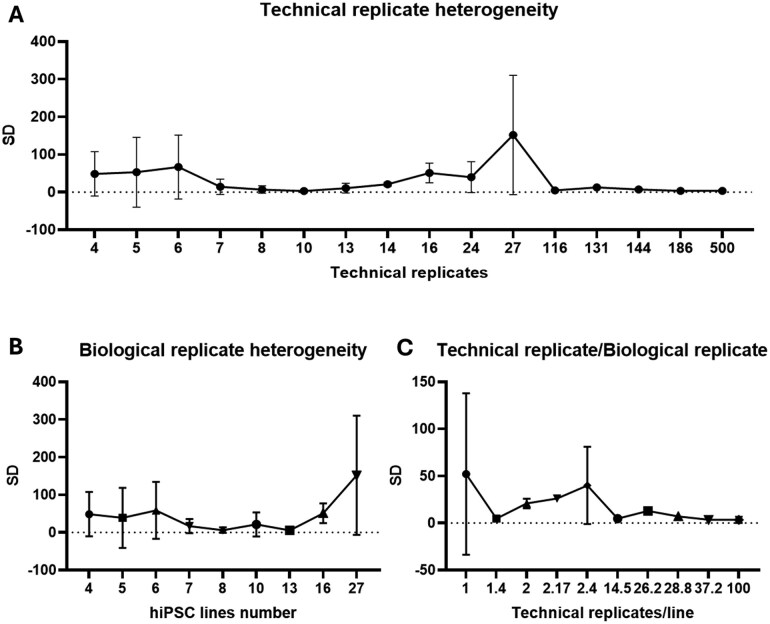
Heterogeneity in hiPSC-CM studies. (A) Technical replicate heterogeneity is defined by the SD of all studies vs the technical replicates used. (B) Biological replicates heterogeneity is defined by the SD of all studies per hiPSC line number. (C) Variation in the ratio of technical replicates divided by the biological replicates (hiPSC lines).

Next, we performed a meta-analysis of all readouts that were reported in 2 or more studies and in more than 4 cell lines (Figure 1). The variability of each readout was based on the provided original data from 4 or more independent hiPSC lines for CM purity, sarcomere morphology, contractility, and calcium handling. The percent change was reported for all cardiac toxicity and drug response studies (Figure 2). Finally, to analyze power and to determine representative within-line variances, we used the data variation within our meta-analysis. By calculating the SD within technical replicates, hiPSC lines/study, and technical replicate/line determined a representative range of hiPSC lines (Figure 3).

### Disease modeling

With the recent hiPSC reprogramming technology, creating many patient-derived hiPSC lines is now feasible, thereby representing individual donor genetics. Patient-specific hiPSC-CMs create the possibility of analyzing the molecular mechanism of cardiomyopathies in an early stage before cardiac remodeling or end-stage pathological levels are reached. Most studies describing 4 or more hiPSC lines were reported in disease modeling studies (9). Overall, most disease modeling studies used HCM-related hiPSCs (21 lines), followed by DCM (7 lines), catecholaminergic polymorphic ventricular tachycardia (6 lines), long QT syndrome (4 lines), and Duchenne muscular dystrophy (DMD, 4 lines) vs healthy controls or probands (16 lines). All studies described the pathological and pathophysiological mechanisms of the studied disease. Some studies reported both disease modeling and pharmacological restoration^14^^,^^16^ or accurately classified genetic cardiac diseases by using machine learning algorithms.^12^ Thus, hiPSC-CM models can also be used in personalized medicine, although a variability in disease severity in DMD hiPSC-CMs can change the phenotypic outcome per hiPSC line. For example, it was observed that metabolic activity was only affected in 13-, 32-, or 50-year old patients, but not in hiPSC-CMs from a 7-year old far less sick patient.^14^ Additionally, more studies reported on the heterogeneity between disease mechanisms within the patient population.^17-19^ In summary, the overall efficiency of disease modeling with hiPSC-CMs is high, although variability remains often too high. The reasons for the variability are several-fold, from age/sex^14^^,^^17^^,^^18^ to cell plate localization^15^ protocols and pacing^17^, and disease-specific mutation sites^17-19^ or a combination thereof. Using hiPSC-based models for complex diseases remains challenging, considering that many diseases are often characterized by late onset, involve probably multiple gene variants, and involve even different tissues and cell types. Therefore, it is important to identify the appropriate control lines, for example, healthy probands, or isogenic cell lines to limit genetic variability and ascertain pathogenicity. Human-induced pluripotent stem cell–derived cardiomyocytes should only be used when having similar spontaneous beat rates or when controlled by pacing, and should be used from the same differentiation technique and batch to avoid phenotypic variability that cannot be attributed to pathologic mutations.

### Cardiac readouts and technical variation

The purity of CMs derived from hiPSCs is of great importance for the functional outcomes and interindividual variation. Here, 7 out of 21 studies described the purity of their CM populations, with an average of 67.48±10.9% being CMs (Figure 1A), in which 6 studies used cardiac troponin T as a specific CM marker, whereas 1 study used alpha-actinin.^26^ The technical replicates varied between 1 and 100 samples per line. Sarcomere morphology determined by sarcomere length and sarcomere disorganization revealed a consistent number for length (1.75 ± 0.74 μM) in 3 studies with 36 hiPSC-CM lines, whereas disorganization analysis results in higher viability (16.87 ± 7.86%) in 17 hiPSC-CM lines (Figure 1B). The contractile function of hiPSC-CMs has often been determined by beats per minute (BPM) or force generation (Figure 1C). Force was described by 2 studies and 12 individual hiPSC-CMs by 0.14 ± 0.07 mN. The BPM was extensively provided (5 studies in 12 hiPSC-CMs) and reported an average BPM of 35.92 ± 21.81. Finally, calcium handling was analyzed by percentage of arrhythmogenic transients, amplitude, and action potential duration at 90% repolarization (APD90) (Figure 1D). Arrhythmogenic value was found in 42.07 ± 9.3% of obtained transients in 3 studies with 17 hiPSC-CM lines. Amplitude of 323.63 ± 93.8 mV was described in 4 studies and in 51 hiPSC-CMs in total. In 43 hiPSC-CMs of 4 studies, APD90 of 310.14 ± 116.24 ms was reported. Altogether, the percentage of the SD per readout mean is presented in Figure 1D. Mainly, BPM, contractility, and sarcomeric morphology readouts demonstrated a high heterogeneity, leading to a SD variation of >40%.

### Drug response and cardiac toxicity studies

Drug development for cardiac toxicity is a costly and lengthy process with an alarmingly high failure rate of approximately 90% of identified potential new drugs in the last 30 years.^33^ Due to their scalability, hiPSC-CM models have been established for HT screenings for drug response or cardiac toxicity testing. Several successful screens are outlined in Table 1. Here, we summarized the percentage of change, which is the relative change from baseline to the treated condition in healthy subject hiPSC-CM lines. On average, studies have used 10 hiPSC lines with 61.4% of change compared to baseline (Figure 2). Interestingly, we observed that SD of these studies is 66%, meaning that the variation in change toward baseline is greater than the actual percentage of change.

Subsequent HT hiPSC-CM studies using therapeutically relevant drug concentrations with paced or unpaced conditions demonstrated >70% in sensitivity and specificity scoring.^22^^,^^34^ By using 8 commercially available healthy subject hiPSC lines, drug effects resulted in an overall response rate of 87.9% of canonical drug responses in the engineered heart tissue (EHT) model.^21^ Correctness scoring was determined by the precision and accuracy of a readout to provide the correct results or effects as required. Here, the correctness score varied between 80% (1 cell line) and 92%-93% (3 cell lines), suggesting that differences in baseline EHT contraction might be less relevant for drug screening, but at least 2 cell lines would be necessary to increase the precision of the assay >80% and detect all drug effects. The variability confounding dose–response curves could be explained by the high level of baseline variability between cell lines observed in many studies described in Table 1. For example, the viability in the EHTs of 10 healthy controls was linked to proprietary differences in the culture conditions of commercial parties.^21^ Also, dose–response variability was observed in female vs male hiPSC donors.^23^ Other studies have reported higher variation of drug response per line, with torsadogenic reagents causing early after depolarizations (EADs) in 2 female lines, but none in all 3 male lines tested.^23^ Additionally, 1 line did not show a chronotropic effect to isoproterenol. Another study showed various responsiveness in 2 lines, showing no EADs to quinidine but a high number to moxifloxacin (Mox)^24^ and authors concluded that not all hiPSC-CMs are suitable for drug testing in terms of arrhythmogenic safety assessment.

The validation of 23 cardiotoxic-reported drugs described a good correlation between *in vitro* hiPSC-CM–observed cardiotoxicity and the clinical incidence.^26^^,^^27^^,^^29^^,^^32^ One study described that 27 hiPSC-CM line can be used as well to characterize inter-individual responses in untreated and cardiotoxic drugs–treated hiPSC-CMs.^30^ In contrast to this finding, the overlap between the drug responses to dofetilide and Mox in 16 hiPSC-CM cell lines of both compounds and the corresponding subject–derived hiPSC-CMs failed to find a significant correlation.^28^ This suggests that to study cardiotoxicity, more than 16 individual hiPSC lines need to be considered.

Together, the growing utilization of hiPSC models in toxicity and drug screenings validates their reliability and fidelity, supporting their potential as a platform for conducting “CTiD.” Considering that hiPSCs may replicate human diseases, further advancements in these areas should open the door for next-generation drug development strategies.

### Heterogeneity and statistical power consideration

As described above, many hiPSC-CM studies suffer from variations in disease phenotype, toxicity correlation, and drug responses. To illustrate how heterogeneity affects the SD, we calculated the effect of variability per hiPSC-CM study to detect differences in the readouts between cases and controls (Figure 3). Heterogeneity between cells of different subjects within each group is expressed in SDs. We used the reported variation per technical replicate number to determine the within-cell-line variances (Figure 3A). Here, we observed mainly an effect in total SD when the reported study SD is also increased, indicating a technical SD heterogeneity, rather than a higher statistical power when using more technical replicates. Next, we plotted the SD per biological replicate to determine the within-cell-line variance (Figure 3B). Again, the highest effect is observed in the hiPSC line SD, with even higher heterogeneity in 16 or 27 hiPSC lines. Finally, we report the relative heterogeneity as the ratio between the within-group SD and the mean difference between the groups. Here, a major SD was found in studies that only used 1 technical replicate per hiPSC-CM line (Figure 3C). Overall, we conclude that the larger this heterogeneity, the larger the required sample size becomes. Ideally, the SD within each group is much smaller than the SD between the groups. However, since we cannot control the effect sizes of the biological readout (ie, the difference in the measured cellular phenotype between hiPSC lines and controls), it would be advisable to determine and reduce variability of the desired *in vitro* readouts within one hiPSC-CM group.

## Discussion

Biological heterogeneity is a natural phenomenon that plays a role in cardiovascular research. Still, many hiPSC-based cardiovascular studies published over the past decade are based on very limited cohort sizes. In this review, we summarized the 21 studies describing cardiac outcomes between more than 4 hiPSC lines. Despite the improved standardized CM generation protocols and QC, variability at baseline level readouts is apparent, including when comparing commercially available cardiomyocyte cell lines. Many cardiac readouts have shown heterogeneity among hiPSC “line-to-line” and technical replicates or so-called “batch-to-batch variation.” For example, compiling and comparing action potential duration (APD) data from 18 academically and commercially available lines revealed a variation of ∼400% between lines (120-600 ms), far exceeding the 50% variability in the normal QT interval range for human hearts.^35^ In our obtained dataset, APD90 ranged from 250 to 341.8 ms, with an average variation of 37% between lines and studies. We reported large baseline differences in contractile force and kinetics (>49%). Additionally, a high heterogeneity in cardiomyocyte morphology (>42%) and metabolism (>5-fold)^35^ has been observed, which exceeds the pathological impact caused by sarcomeric mutations associated with, for example, HCM. After these observations, we discuss 3 categories to explain the heterogeneity found in hiPSC-based cardiovascular research.

First, the variability could be impeded by the baseline variability caused by donor-related factors (eg, hiPSC line genetic background, human leukocyte antigen [HLA] haplotype, sex, age, and health status). The bigger an effect size, the lower the number of independent hiPSC lines and total observations needed to reach sufficient statistical power. Therefore, the recapitulation of the prediction in hiPSC-CM models could be impeded by the baseline differences in the hiPSC. In this review, various studies have attempted to avoid donor-related factors. For example, Zaunbrecher et al. used CRISPR/Cas9 to generate homozygous truncations in the Z disk and A-band of the titin gene, avoiding multiple patient-specific backgrounds.^19^ Next, although the HLA complex is associated with population-specific adverse drug reactions, no hiPSC population differences in drug responses or toxicity are determined due to the lack of cohorts of HLA-defined hiPSCs.^31^ Moreover, dose–response variability was observed in female vs male hiPSC donors.^23^ Finally, data would suggest that hiPSC donor age is associated with an increased risk of abnormalities in hiPSCs due to both epigenetic and genetic aberrations that increase with age.^36^ Nevertheless, the generalizability of these study designs is restricted. Instead, using several isogenic pairs increases the power up to 60% or requires up to 5-fold fewer lines, while allowing generalization of the findings to the larger patient population.^37^ The isogenic approach might be more feasible, given that a wide selection of lines for some rare diseases might be difficult. Previous reports and our overview clearly indicate that “unrelated control lines” are unsuitable for disease modeling studies and confirm the request for isogenic controls to substantiate often subtle phenotypic differences between mutation-carrier and non-carrier lines. On the other hand, drug response and toxicity heterogeneity were found to be relatively low, which indicates that healthy control lines could be used for this line of screening. For example, hiPSCs from donors with different susceptibilities to Mox-induced QT prolongation were observed in the hiPSC-CMs from each individual, suggesting that hiPSC-CMs successfully predict susceptibility to Mox-induced QT prolongation and clinical outcomes.^26^ Still, using more than one isogenic control per study should be considered. Thus, by selecting genetically homogeneous donor cases and controls, the study’s intrinsic cellular variance can be lowered, which is a critical determinant in both increasing statistical power and evaluating results from hiPSC studies for important findings.

Second, process-related variability such as differences in reprogramming methods, differentiation protocols, purity, and culture conditions could be contributing to heterogeneity among hiPSC line outcomes. The impact of hiPSC-based studies on scientific progress has likely not yet reached its maximum potential, as considerations regarding the choice and optimization of study designs remain often not reported. In our systematic search, 21 studies were found to report 4 or more hiPSC lines of the same condition, whereas many of them only reported no individual values in the text or figures, or only the quality controls per hiPSC-CM line. As described in Table S1, we found a high variability in the reprogramming methods, differentiation protocols, and coating culture conditions of these studies. For example, many hiPSC lines were reprogrammed from fibroblasts or PBMCs, but adipose, epithelial, dental, cord blood or embryonic stem cells were also used to generate hiPSC lines, and even different cell types were used within one study. As summarized by Lee et al., cardiac fibroblast–derived hiPSC lines revealed different field potential durations, higher APD/conduction velocities, and more mature ion channels and calcium handling protein levels compared to hiPSC derived from dermal fibroblast of blood.^38^ Next, we found a high viability in the differentiation protocols. Some studies even used different protocols or commercial protocols that did not specify differentiation details, which hinders rigor and reproducibility and reduces translational value. Currently, most cardiac differentiation protocols are based on programmed activation and then inhibition of the Wnt signaling pathway, with many variants thereof (reviewed by Maas et al.^39^) Differentiation and culture formats differ as well. Human-induced pluripotent stem cell can be generated via co-culture with mouse visceral endoderm–like (END-2) stromal cells, EB differentiation in suspension, and monolayer differentiations, each with different CM yield, maturity, and cost advantages. Overall, monolayer differentiations are currently most used due to their higher reproducibility and robustness. The coating medium for CMs can vary as well, which influences the functional properties and maturation of CMs. Matrigel was found to produce more mature CMs with higher dV/dt_max_, increased potassium current densities, and the presence of cardiac troponin I.^40^ Next, to avoid high variability in basic functionality of cardiomyocytes, the CM purity needs to be evaluated at the time when the culture is ready for disease modeling or pharmacological and toxicological interventions. We summarized the purity percentages of 7 studies and found an average of 67.48±10.9%. Some studies described relatively low purity samples of <55%, which corresponded with the EB differentiation protocol.^15^^,^^16^^,^^27^ Higher purities (>80%) were described with the monolayer differentiation protocol,^19^^,^  ^25^^,^^26^ indicating a more robust and more efficient differentiation strategy. Despite a few, many studies in Table 1 did not specify the CM purity or quality control measures taken before experiments. Finally, although convenient, 2D cell culture techniques have unfortunately proven to be ineffective in mimicking the contractile deficit in titin patient–derived hiPSC-CMs. Instead, the titin-mutant hiPSC-CMs in EHT models showed the obvious insufficient contractile force of the patient-derived hiPSC-CMs.^41^ The limited predictivity in the 2D hiPSC-CM models poses the question if these methods can yield cells with enough phenotype, in a realistic, large-scale, and financially appropriate culture time frame. Implementation of standard operating procedures, including a unified differentiation protocol, coating material, culture conditions, and quality controls, may reduce variation within and between hiPSC lines, thus improving attainable power. As the field progresses, combining and optimizing culturing and differentiation protocols, long-term culturing, 3D tissue engineering such as EHTs, cardiac organoids, or organ-on-chip technologies, and metabolic supplementation (reviewed elsewhere)^5^^,^^34^^,^^42^^,^^43^ could take us to the final steps in getting as close to the best model fit for purpose; as simple as possible, yet, as complex as necessary.

Third, intrinsic cellular differences such as variability in epigenetic states, gene expression profiles, and functional maturity of derived cardiomyocytes are discussed. The individual genetic variation in ion channels, sarcomeric protein expression, and drug response in healthy control hiPSC-CM supports the request for isogenic controls or multiple hiPSC clones. Additionally, using singe-cell sequencing or *in silico* tools like artificial intelligence (AI) could be implemented to distinguish between the individual genetic variation and phenotype, drug response, or toxicity. Grimm et al.^30^ described that the high inter-individual variability between 27 donors was the dominant contributor to overall variability for the peak frequency (27% Coefficient of Variation between donors vs 28% total Coefficient of Variation), decay/rise time (10% vs 11%), and peak amplitude (10% vs 13%) with very little contribution from process variability. Maturity is a big factor for CM function and morphology and despite considerable progress, hiPSC-CMs are still far from expressing adult-like phenotypes *in vitro*. The age reported in the studies of Table 1 ranged between day 13 and day 60 after differentiation, indicating a high difference in maturity status. Using biochemical strategies such as metabolic maturation and prolonged culture time^44^ or biophysical strategies such as matrix, stretch, or 3D culturing^45^ could significantly improve hiPSC-CM maturity. Variation within a specific readout should be considered because higher readout variability results in lower statistical power for the same mean difference and sample size. As described above, some readouts result in an overall SD of >40% among studies which can lead to a higher intrinsic variability vs the studied effect. Here, machine learning and AI could enable the assessment of changes, facilitating the detection of disease modeling, drug efficacy, and adverse effects. Finally, hiPSC-CM studies require different statistical approaches and different sample sizes to obtain adequate statistical power. Statistical power is affected by the pattern of means: including groups with varying effect sizes negatively impacts power to detect an overall effect, while including multiple groups that are expected to show the same experimental effect size does not affect power. Unfortunately, “power failure” is a general and ubiquitous problem in cardiovascular research. In this study, limitations arise due to study design of the described studies (eg, variability in protocols, sample sizes, and widely used standardized readouts). Potential biases can also exist due to combining data values of various studies and by comparing studies with disparate methodologies. We found that our meta-analyses indicate that most hiPSC-CM studies across various readouts result in inadequate statistical power. As an example, the study of Carvalho et al.^17^ revealed high variability of arrhythmias per hiPSC-CM line, per differentiation protocol (EB vs 2D), and batches. Even with this high sample size (*n* = 642 samples), the arrhythmia readout resulted in inadequate statistical power. Many studies described variability in drug response or no significant correlation to the clinical response, which could be related to the use of one technical replicate per hiPSC line.^23^^,^^24^^,^^28^^,^^30^^,^^32^ A better result was found in the study of Mannhardt et al.,^21^ where drug responses were qualitatively similar for each healthy donor. Here, 116 technical replicates from 10 hiPSC-CM lines in 3D format were used. Suboptimal study design choices, underpowered studies, and/or incorrect statistical analyses all reduce the chance of detecting true effects and bias the estimates of true effects, making correct statistical analysis crucial for the optimal utilization of hiPSC-based cardiovascular research. In summary, the usage of isogenic controls, rather than probands or unrelated healthy controls, low variability in technical replications, and standardization in protocols will improve statistical power of hiPSC-CM studies.

## Conclusion

Each hiPSC-CM model is as unique as the individual from whom it was derived, along with a large amount of known and unknown experimental heterogeneity. Although every effort should be made to understand and reduce readout heterogeneity, a more immediate strategy would be for each hiPSC modeling community to adopt a set of appropriate common case and control lines that would enable them to identify experimental variation across studies. Taken together, hiPSC-CM models are more complicated than the present *in vitro* testing approaches, and optimization is in many ways a study itself, demanding its unique protocols, robotics, standardization, and automated data analysis. Given the above, it can be assumed that hiPSC-CM models could eventually be included in regulatory documents as the first CTiD screen before drug evaluation in patients. Next, the combination of the clinical findings with the CTiD data with machine learning algorithms could help the clinical choices of care by cardiologists. Moreover, eventually, HT analysis of hiPSC-CMs holds promise of reducing the need for *in vivo* studies, although the secondary effects in pharmacodynamic, tissue interaction, and metabolite activity remain challenging to study *in vitro*. Study designs using specific readouts or different cell types may yield different statistical powers and thus may require researchers to perform their own pilot studies to improve their study design. With rigorous study designs and appropriate statistical analyses, hiPSC technology can significantly reduce data heterogeneity and advance scientific progress in cardiovascular research.

## Supplementary Material

szag001_Supplementary_Data

## Data Availability

No new data were generated or analyzed in support of this research.
