# Supplementary material for: Heterogeneity and optimal study design between cell lines in induced pluripotent stem cell-based cardiac disease modeling
Source: Stem Cells Transl Med. 2026 Jan 28;15(2):szag001. doi: 10.1093/stcltm/szag001 (PMC12866806; doi:10.1093/stcltm/szag001)
Supplement: szag001_Supplementary_Data [file szag001_supplementary_data.docx]

Supplementary Information

**Supplementary Information:**

**Heterogeneity and Optimal Study Design between cell lines in hiPSC-based Cardiac Disease Modelling**

Renee G.C. Maas1,2, Chris Denning3, Joost P.G. Sluijter1,2

1Utrecht Regenerative Medicine Center, Circulatory Health Research Center, University Utrecht, 3584 CS Utrecht, The

Netherlands

2Department of Cardiology, Experimental Cardiology Laboratory, University Medical Center Utrecht, Heidelberglaan 100, 3584

CX Utrecht, The Netherlands

3Department of Stem Cell Biology, Biodiscovery Institute, University of Nottingham, University Park, Nottingham, NG7 2RD, UK.

Supplementary Table 1. Overview of cell culture strategies used in Table 1.

| **Study type** | **hiPSC lines (number)** | **CM**  **Age (Days)** | **hiPSC source** | **Differentiation protocol** | **Coating** | **ref.** |
| --- | --- | --- | --- | --- | --- | --- |
| Disease modelling | RYR2 (6), TPM1 (2), MYBPC3 (2), KCNQ1(2), HERG (4), LMNA(2), Healthy subjects (2) | ~30-50 | Fibroblasts | EB: hiPSC–END-2 cocultures, mitomycin C, DMEM + 10% FCS | Gelatin | [[12]](https://paperpile.com/c/YLR5ZB/Ldin) |
| Disease modelling | MYBPC3 (4), Healthy  subject (1) | 13 | PMBCs | EB: RPMI/B27+ CHIR, IWP-4 | Matrigel | [[13]](https://paperpile.com/c/YLR5ZB/rSh2) |
| Disease modelling | DMD (4), Healthy subjects (2) | ~20 | Fibroblasts | 2D: RPMI/B27+ CHIR, IWP-4 | Matrigel | [[14]](https://paperpile.com/c/YLR5ZB/OBmb) |
| Disease modelling | TNNT2 (4), Probands (3) | 20-50 | Adipose stem cells | EB: BMP4, hβFGF, activin A and DMEM + 10% FCS | Gelatin | [[15]](https://paperpile.com/c/YLR5ZB/tAWB) |
| Disease modelling | MYH7 (5), Probands (5) | 20-60 | Fibroblasts | EB: BMP4, hβFGF, activin A and DMEM + 10% FCS | Gelatin | [[16]](https://paperpile.com/c/YLR5ZB/K76r) |
| Disease modelling | Healthy subjects (6) | 30 | 1x hESC, 5x PBMCs | 1x EB: StemPro-34+ BMP4, Activin A and bFGF; XAV939  5x 2D: RPMI/B27+ CHIR99021; XAV939 | Matrigel | [[17]](https://paperpile.com/c/YLR5ZB/WSeT) |
| Disease modelling | LMNA variants (7),  healthy subjects (3) | ~40 | PBMCs | 2D: STEMdiff Cardiomyocyte Differentiation Kit and RPMI+B27 | Matrigel | [[18]](https://paperpile.com/c/YLR5ZB/uB9M) |
| Disease modelling | Healthy subject (1), iTTN (4) | ~40 | Fibroblasts | 2D: RPMI/B27+ Activin A, BMP4, CHIR, and XAV939 | Matrigel | [[19]](https://paperpile.com/c/YLR5ZB/My4I6) |
| Disease modelling | MYBPC3 (1), MYH7 (4), DMD(3),  TNNT2(3), TTN(2), Healthy subjects (5) | 30 | PBMCs | 2D: RPMI/B27+ CHIR, IWR-1 | Matrigel | [[20]](https://paperpile.com/c/YLR5ZB/Dnp2F) |
| Drug response | Healthy subjects (10) | ~50 (EHTs) | 6x Fibroblasts, 1x epithelial, 1x dental pulp, 1x hESC, 1x cord blood | Commercial or EB: RPMI/B27+ BMP4, Activin A, bFGF | N/A, EHTs | [[21]](https://paperpile.com/c/YLR5ZB/dYIx) |
| Drug response | RyR2 (6) | ~30-50 | Fibroblasts | EB: hiPSC–END-2 cocultures(12742992), mitomycin C, DMEM + 10% FCS | Gelatin | [[22]](https://paperpile.com/c/YLR5ZB/9xhO) |
| Drug response | Healthy subjects (6) | ~25 | PBMCs | Commercial | Gelatin | [[23]](https://paperpile.com/c/YLR5ZB/E1G8) |
| Drug response | Healthy subjects (5) | ~25 | PBMCs | Commercial | Fibronectin | [[24]](https://paperpile.com/c/YLR5ZB/ud52) |
| Cardio- toxicity | Healthy subjects (6) | 27-29 | Fibroblasts | 2D: RPMI/B27+CHIR, WNT-C59 | Matrigel | [[25]](https://paperpile.com/c/YLR5ZB/7uwA) |
| Cardio- toxicity | Healthy subjects (10) | ~40 | Fibroblasts | 2D: StemPro34+ Blebbistatin, BMP4, Activin A, IWR-1 | Fibronectin | [[26]](https://paperpile.com/c/YLR5ZB/fkQg) |
| Cardio- toxicity | Healthy subjects (14) | ~50 | Fibroblasts | EB: StemPro-34+ BMP4, Activin A and bFGF; XAV939 | Matrigel | [[27]](https://paperpile.com/c/YLR5ZB/ixgf) |
| Cardio- toxicity | Healthy subjects (16) | ~30 | PBMCs | Commercial | Matrigel | [[28]](https://paperpile.com/c/YLR5ZB/64C0) |
| Cardio- toxicity | Healthy subjects (11) | ~40 | Fibroblasts/PBMCs | 2D: CDM3+CHIR, WNT-C59 | Matrigel | [[29]](https://paperpile.com/c/YLR5ZB/WTnW) |
| Cardio- toxicity | Healthy subjects (27) | ~40 | PBMCs | Commercial | Gelatin | [[30]](https://paperpile.com/c/YLR5ZB/oJzL) |
| Cardio- toxicity | Homozygous HLA donors (13) | 48 | PBMCs | 2D: RPMI/B27+CHIR, IWR-1 | Matrigel | [[31]](https://paperpile.com/c/YLR5ZB/rmZn) |
| Cardio- toxicity | Covid (13) | n/r | PBMCs | 2D: RPMI/B27+CHIR, IWR-1 | Matrigel | [[32]](https://paperpile.com/c/YLR5ZB/FnLP) |

Table abbreviations: DMD; Duchenne muscular dystrophy, EB; Embryonic body formation , EHTs; engineered heart tissues, hESC; human embryonic stem cells, HLA; human leukocyte antigens, KCNQ1; potassium voltage-gated channel subfamily Q member 1, LMNA; lamin A/C, MYBPC3; myosin-binding protein C, MYH7; myosin heavy chain 7, PBMCs; Peripheral Blood Mononuclear Cells , RYR2; Ryanodine receptor 2, TNNT2; cardiac troponin T; TPM1; tropomyosin 1, TTN; titin, 2D: 2-dimentinal monolayer differentiation.
